# Treating Unmet Needs in Psychiatry (TUNE-UP): Developing a Novel Service for Individuals With Psychosis With Refractory Cognitive, Negative, and Positive Symptoms

**DOI:** 10.1192/bjo.2024.520

**Published:** 2024-08-01

**Authors:** Ioana Varvari, Valentina Mancini, Chambrez Zauchenberger, Phillip McGuire, Robert McCutcheon

**Affiliations:** 1 Department of Psychiatry, University of Oxford, Oxford, United Kingdom; 2 Oxford Health NHS Foundation Trust, Oxford, United Kingdom; 3 Department of Psychosis Studies, Institute of Psychiatry, Psychology and Neuroscience, King's College, London, United Kingdom; 4 Department of Psychiatry, Oxford University, Oxford, United Kingdom

## Abstract

**Aims:**

While dopamine antagonists are an effective treatment for positive psychotic symptoms, they are rarely effective when it comes to treating the cognitive (memory, learning, planning, etc.) and negative (avolition and social withdrawal) symptoms of the disorder. Furthermore, for a sizeable proportion, standard dopamine antagonists are not effective for positive symptoms either. As such, refractory symptoms are a major burden for patients, carers, and clinical services.

**Methods:**

To address this, The TUNE-UP (Treating Unmet Needs in Psychiatry) clinic in Oxford was established in September 2023 as an innovative solution aiming to: (A) Undertake an in-depth assessment of cognitive, negative, and positive symptoms; (B) Identify potentially modifiable causative factors contributing to refractory symptoms (e.g., cholinergic burden, sleep disturbances, physical comorbidities, affective symptoms); and (C) Implement management plans including community clozapine initiation where appropriate. We have analysed data from the clinic's initial five months of operation to establish a baseline understanding of our patient population and identify trends in symptoms.

**Results:**

In the first five months of operations, 21 referrals were accepted comprising 80.9% males (mean age 43.3 years, SD 13.7). 3 were referred for cognitive symptoms, 1 for negative and cognitive symptoms, 11 for positive symptoms, 3 for medication optimisation, and 3 for clozapine re-titration. Of those fully assessed (N = 17), mean total symptom scores measured using the Positive and Negative Syndrome Scale (PANSS) were of mild/moderate severity (70.5, SD 18.4). Objective cognitive testing via the Screen for Cognitive Impairment in Psychiatry (SCIP) demonstrated a total mean score of 54.1 (SD 12.1), markedly below what would be expected in a matched control population (76.3). Cognitive scores were lower in those of older age (r = −0.62, p = 0.01). Subjective experience of cognitive impairment was measured using the Subjective Scale to Investigate Cognition in Schizophrenia, poor subjective cognition was associated with more severe negative symptoms (r = 0.57, p = 0.03), but not objective SCIP results (r = 0.12, p = 0.85).

**Conclusion:**

Refractory positive symptoms remain a priority for clinicians, but cognitive and negative symptoms are highly prevalent reinforcing the need for a comprehensive approach. Routine structured assessment of all symptom domains is feasible in clinical practice. Future work should examine the longitudinal impact of various interventions on different symptom domains.

